# Molecular regulations of circadian rhythm and implications for physiology and diseases

**DOI:** 10.1038/s41392-022-00899-y

**Published:** 2022-02-08

**Authors:** Francesca Fagiani, Daniele Di Marino, Alice Romagnoli, Cristina Travelli, Davide Voltan, Lorenzo Di Cesare Mannelli, Marco Racchi, Stefano Govoni, Cristina Lanni

**Affiliations:** 1 Department of Drug Sciences (Pharmacology Section), University of Pavia, V.le Taramelli 14, 27100 Pavia, Italy; 2 Department of Life and Environmental Sciences, Polytechnic University of Marche, via Brecce Bianche, 60131 Ancona, Italy; 3 New York-Marche Structural Biology Center (NY-MaSBiC), Polytechnic University of Marche, via Brecce Bianche, 60131 Ancona, Italy; 4 NEUROFARBA Department, University of Florence, Florence, Italy

**Keywords:** Cell biology, Inflammation, Cancer

## Abstract

The term “circadian rhythms” describes endogenous oscillations with ca. 24-h period associated with the earth’s daily rotation and light/dark cycle. Such rhythms reflect the existence of an intrinsic circadian clock that temporally orchestrates physiological processes to adapt the internal environment with the external cues. At the molecular level, the circadian clock consists of multiple sets of transcription factors resulting in autoregulatory transcription-translation feedback loops. Notably, in addition to their primary role as generator of circadian rhythm, the biological clock plays a key role in controlling physiological functions of almost all tissues and organs. It regulates several intracellular signaling pathways, ranging from cell proliferation, DNA damage repair and response, angiogenesis, metabolic and redox homeostasis, to inflammatory and immune response. In this review, we summarize findings showing the crosstalk between the circadian molecular clock and some key intracellular pathways, describing a scenario wherein their reciprocal regulation impinges upon several aspects of mammalian physiology. Moreover, based on evidence indicating that circadian rhythms can be challenged by environmental factors, social behaviors, as well as pre-existing pathological conditions, we discuss implications of circadian misalignment in human pathologies, such as cancer and inflammatory diseases. Accordingly, disruption of circadian rhythm has been reported to affect several physiological processes that are relevant to human diseases. Expanding our understanding of this field represents an intriguing and transversal medicine challenge in order to establish a circadian precision medicine.

## The rhythm around the molecular clock

The term circadian rhythm was originally coined by Halberg to indicate the near-24-hour (h) endogenous oscillations of biological processes in organisms associated with the earth’s daily rotation cycle^1^. Such endogenous rhythms, observed in organisms ranging from photosynthetic prokaryotes to higher eukaryotes, reflect the existence of an intrinsic circadian clock that temporally orchestrates physiological and behavioral processes^2^ with the specific function of coordinating and adapting the internal environment with the external cues^3^. The entrainment of circadian rhythms, which consists of the alignment of the endogenous circadian oscillator to external stimuli, relies on external cues, such as the light pattern and food intake. In particular, the daily light–dark cycle represents the primary external synchronizer of circadian rhythms. In mammals, light is processed through the eye and transmitted through the retinohypothalamic tract to hypothalamic suprachiasmatic nucleus (SCN), the master internal pacemaker. In the retina, the intrinsically photoreceptive retinal ganglion cell (ipRGC) expressing the photopigment melanopsin, which renders them photosensitive to short-wavelength irradiation, together with the retinal rod and cone photoreceptors, conveys photic information to entrain SCN clocks^4^. Notably, SCN is composed of bilateral nuclei containing approximately 10,000 neurons, each of them displaying a cell-autonomous circadian oscillator. A number of papers have demonstrated that the SCN is both necessary and sufficient for the generation of circadian rhythms in rodents^5^. SNC neurons have distinct and topographically organized coupling mechanisms that allow them to remain synchronized to one another. They generate a pronounced circadian rhythm of neuronal firing frequency, which allows them to synchronize other cells throughout the body.

Notably, in the brain, beside the master clock in SCN operating as self-sustaining clocks, other functional nuclei have been found to act as semiautonomous clocks (i.e. olfactory bulb, dorsomedial hypothalamus, arcuate nucleus, habenula) or as slave oscillators (i.e. bed nucleus of the stria terminalis, amygdala, preoptic area, paraventricular nucleus, nucleus accumbens), both coordinated by the SCN.

The central pacemaker clock synchronizes multiple peripheral clocks expressed in nearly every mammalian organ (i.e. such as lungs, liver, heart, and skeletal muscle). Noteworthy, while in the SCN circadian rhythms are similar between diurnal and nocturnal species, a phase shift, matched to their active period, in the rhythms has been observed in their peripheral tissues^6^. The synchronization of peripheral clocks is not only hierarchically and vertically controlled by the hypothalamic master clock through the peripheral nervous system, but it also horizontally achieved through both humoral and non-humoral pathways. In particular, SCN controls peripheral oscillators through the autonomic innervation of peripheral tissues, endocrine signaling (glucocorticoids), body temperature, and feeding-related cues. The neural control of peripheral oscillators requires both sympathetic and parasympathetic pathways^7^. SCN projections, through the paraventricular nucleus–superior cervical ganglia (PVN-SCG) pathway, deliver the entraining signal for the submandibular salivary glands^7,8^. Autonomic pathways derived from the SCN provide photic information to oscillators in the adrenal gland and liver^9^. Moreover, sympathetic pathways modulate the sensitivity of the adrenal to adrenocorticotropic hormone (ACTH) and the release of glucocorticoids^9,10^. In the adrenal cortex and medulla, cellular oscillators respond to neural inputs deriving from the SCN^9,11^. Furthermore, glucocorticoids are humoral entraining signal for peripheral clocks entraining signals for peripheral oscillators. In particular, since glucocorticoids-response elements (GREs) are present in promoter regions of the core clock components glucocorticoids regulate the transcriptional activation of clock genes and clock-related genes^12–14^.

### The circadian autoregulatory feedback loop

At the molecular level, the circadian clock consists of multiple sets of transcription factors resulting in autoregulatory transcription-translation feedback loops (TTFLs) that represent the core mechanism of the circadian clock in mammals. A central role in the regulation of this loop is played by the heterodimeric partnership between two transcription factors, i.e. the brain and muscle Arnt-like protein 1 (ARNTL, also known as BMAL1) and the circadian locomotor output cycles kaput (CLOCK). In particular, transcription of *BMAL1* and *CLOCK* genes, or its related gene *NPAS2* (neuronal PAS domain containing protein-2), which is mainly expressed in the forebrain, leads to the heterodimerization in the cytoplasm of the BMAL1:CLOCK complex, which translocates into the nucleus where it binds to canonical Enhancer Box (E-Box)-sequences containing the consensus sequence CACGTG or noncanonical E-Boxes of clock-regulated genes. Among the different target genes, BMAL1 and CLOCK also promote the expression of the components of the negative arm of the molecular clock, such as period (*PER1, PER2, PER3*) and cryptochrome (*CRY1, CRY2*). PER and CRY form a complex in the cytoplasm that translocates into the nucleus. Following their translation and nuclear accumulation, PER and CRY inhibit the transcriptional activity of BMAL1:CLOCK complex. At post-transcriptional level, the stability of PER and CRY proteins is regulated by Skp1-Cullin-F-box protein (SCF) E3 ubiquitin ligase complexes, involving β-TrCP (β-Transducin Repeat Containing E3 Ubiquitin Protein Ligase) and F-Box and Leucine Rich Repeat Protein 3 (FBXL3), respectively. The casein kinase 1ε/δ (CK1ε/δ) and adenosine 3’,5’-monophosphate (AMP) kinase (AMPK) phosphorylate PER and CRY proteins, respectively, thus promoting polyubiquitination by their respective E3 ubiquitin ligase complexes, which tag PER and CRY proteins for degradation by the 26 S proteasome complex. Decrease in PER and CRY protein levels relieves the suppression of BMAL1:CLOCK activity, thereby permitting to establish a new oscillatory cycle. In addition to such core loop, further key regulators of the circadian clock, such as the nuclear receptors REV-ERBα (also known as nuclear receptor subfamily 1, group D, member 1, NR1D1) and REV-ERBβ (also known as nuclear receptor subfamily 1, group D, member 2, NR1D2) (REV-ERBs), as well as the retinoic acid orphan receptor (ROR) (RORα, RORβ, and RORγ) establish another feedback loop. In particular, while REV-ERBs act as transcriptional repressors of *BMAL1* expression, RORs positively regulate the expression of BMAL1 by binding to sites Retinoic acid receptor-related Orphan Receptor Element (RORE) elements in the *BMAL1* gene promoter.

On the other hand, the biological clock is not only based on transcriptional mechanisms, but also, membrane depolarization, intracellular calcium flux, and activation of cyclic AMP (cAMP) signaling appear to be important regulators of the mammalian transcriptional clock. Accordingly, intracellular calcium is fundamental for neuronal firing rhythms in SCN slices^15^ and sufficient membrane depolarization, periodic calcium influx, and daily activation of cAMP signaling are required for the rhythmic expression of the core clock components in SCN neurons^16–18^. Notably, such effects are mediated by the phosphorylation-dependent activation of the calcium/cAMP response element binding protein (CREB) that binds to calcium/cAMP regulatory elements (CREs) on DNA. In particular, CRE sequences have been found in the promoters of several clock genes, such as *PER1* and *PER2*^18,19^. Since membrane potential^20^, calcium flux^15^, and activation of cAMP signaling^16,18^ have been reported to be also rhythmic themselves in SCN, they represent both outputs of and inputs to the transcriptional clock, by possibly establishing positive feedback loops participating in rhythm generation.

However, the mechanisms underlying the molecular clock cannot be simplified in such a reductionist fashion. Indeed, clock genes integrate a plethora of different signals to produce an integrated output over the 24-h cycle, in preparation for the diverse tasks related to periods of light or darkness, wherein gene expression changes in a non-linear manner during the transition hours between activity and rest periods with a gating system enhancing or softening signal transduction to avoid interferences of misaligned cycles^21^.

Notably, endogenous cellular clocks drive the rhythmic expression of genes. In particular, approximately up to 20% of the genome is under circadian regulation^22–24^. Accordingly, by assessing the transcriptome atlas of a primate across the major tissues and brain regions, Mure et al. demonstrated that, in baboons, daily expression rhythms occurs in >80% of protein-coding genes, responsible for different biochemical and cellular functions, and that such rhythmic activity represents the largest regulatory mechanism capable to integrate diverse biochemical functions within and across cell types. Moreover, 82.2% of genes coding for proteins that are identified as druggable targets by the US Food and Drug Administration display cyclic oscillations in transcription^6^.

## Circadian regulation of physiological processes: reciprocal signaling between the clock and other regulatory networks

In addition to their primary role as generator of circadian rhythm, the biological clock serves a key role in controlling physiological functions of almost all tissues and organs in two major ways. First, central outputs from the SCN and/or local outputs from cell-autonomous peripheral oscillators drive circadian rhythms in several physiological pathways in a rhythmic fashion. Second, the core clock components have been observed to act as key molecular players in many intracellular pathways, serving additional physiological roles. Examples of circadian-regulated physiological pathways includes cell growth, DNA repair and damage, angiogenesis, apoptosis, metabolism, redox state, as well as immune and inflammatory processes^25–28^. Thus, in the following sections, we will critically discuss the mechanistic connection between the circadian molecular clock, which introduces a temporal variable into cellular functions, and other cellular networks and biological pathways.

### Circadian clock and cell cycle machinery

**Fig. 1:**
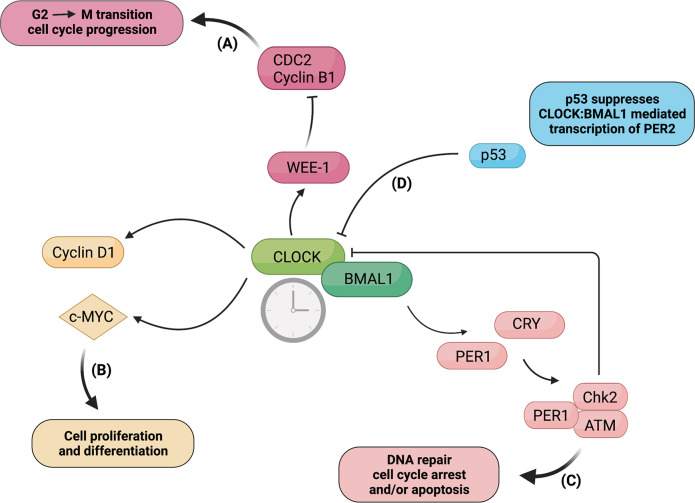
Molecular interaction between the circadian core clock and cell cycle components. The CLOCK:BMAL1 complex transcriptionally activates genes containing E-box regulatory elements in their regulatory regions, such as clock genes and cell-cycle genes. **A** CLOCK:BMAL1 complex directly controls the transcription of the cell-cycle-related gene *Wee-1* contains three B-boxes in its promoter and encodes a protein kinase that inactivates the CDC2/Cyclin B1 complex, thus regulating G2-M transition and cell-cycle progression. **B** Transcriptional activation of the genes encoding Cyclin D1 and C-MYC by CLOCK:BMAL1 affects cell proliferation and differentiation. **C** PER1 can complex with the ATM kinase and the checkpoint kinase Chk2, thus impinging upon DNA repair, cell cycle arrest and/or apoptosis. **D** Both physiological and stress-induced p53 binds to p53 response element in *PER2* promoter, which overlaps with the BMAL1/CLOCK-binding site, thereby inhibiting CLOCK:BMAL1-mediated transcription of PER2. (BioRender.com has been used to create the figure)

Among the diverse biological processes controlled by the circadian clock, the regulation of the cell cycle machinery is the most elusive. However, substantial evidence highlights that the molecular architecture of the circadian oscillator displays several analogies with the complex intracellular machinery regulating cell division and that several regulators of cell cycle progression are expressed in a circadian manner, as elegantly reviewed by Hunt and Sassone-Corsi, 2007. Accordingly, both the circadian clock and the cell cycle are intracellular clocks consisting of sequential phases of transcription-translation and based on the conceptual framework of interlocked autoregulatory loops^29^. Noteworthy, accumulating evidence suggests that the circadian clock affect the timing of cell divisions in vivo^25^. Moreover, most of cell-cycle genes involved in G2-M or G1-S transitions contains E-box regulatory elements in their promoters, transcriptionally activated by CLOCK:BMAL1 heterodimer^29^. Accordingly, Matsuo et al. demonstrated that the cell-cycle-related gene *Wee-1*, which contains three B-boxes in its promoter and encodes a protein kinase that inactivates the CDC2/Cyclin B1 complex, thereby delaying or preventing entry into mitosis, is under the direct transcriptional control of CLOCK:BMAL1^25^. (Fig. 1) The authors found that the diurnal fluctuations in *Wee1* mRNA expression are reflected by its kinase activity, target phosphorylation, and CDC2/Cyclin B1 kinase activity, thus suggesting that the circadian clock–*Wee1* pathway may act as a cell cycle regulator in vivo^25^. Then, another molecular link between clock and cell cycle genes comes from Gréchez-Cassiau et al. work showing that clock genes control G1/S progression by transcriptionally regulating *Cdkn1a*—a gene encoding for the cyclin/Cdk inhibitor p12^WAF1/CIP1^—and that alteration of circadian clock led to aberrant *p21* expression and altered cellular proliferation^30^. Furthermore, CLOCK:BMAL1 complex has been shown to transcriptionally control the genes encoding c-Myc, which regulates cell cycle entry by responding to mitogenic stimuli, and Cyclin D1, thereby modulating cell cycle^31^ (Fig. 1). In this regard, Repouskou et al. demonstrated the in vivo binding of BMAL1 on the human c-Myc promoter and that c-Myc overexpression hindered PER1 transactivation by BMAL1/CLOCK by targeting E-box sequences^31^. The ensemble of this study depicts a scenario wherein the biological clock induces oscillation in Myc expression and, on the other hand, Myc hinders E-box-driven expression of PER1, when its protein abundance allows interaction with BMAL1.

Within this context, the circadian clock has been implicated in DNA damage response (DDR), including cell cycle checkpoints and DNA repair. DNA damage-induced cell cycle checkpoints are signal transduction pathways driven by DNA damage, acting as a genome surveillance mechanism to ensure genomic integrity^32^. Notably, the CLOCK:BMAL1 complex has been found to control DDR by modulating the transcription of genes in genotoxic responses^26^. Moreover, besides a transcriptional regulation, the core clock factors have been also reported to directly interact with key cell cycle checkpoint pathways and, in particular, with DDR factors, thereby maintaining genome integrity. Consistently, PER1 has been reported to complex with the ataxia-telangiectasia-mutated (ATM), a kinase involved in the cellular response to ionizing radiations and DNA double-strand-break-inducing events, as well as with the checkpoint kinases CHK2^33^ (Fig. 1). Moreover, it has been demonstrated that, upon UV damage, CRY1 modulated the ATR (ATM- and Rad3-Related)-mediated DNA damage checkpoint response by interacting with TIMELESS (TIM) in a time-of-day-dependent manner, thereby generating circadian oscillation of ATR activity^34^. Interestingly, Shafi et al. recently demonstrated that androgen receptor-induced *CRY1* was stabilized by genotoxic insult and regulated DDR by directly binding to promoters of homologous recombination factors, thereby modulating genome integrity and promoting castration-resistant prostate cancer growth^27^. As such, CRY1 emerges as a pro-tumorigenic factor that rhythmically controls DNA repair mechanisms and cell survival through temporal transcriptional regulation^27^. Therefore, it is tempting to speculate that failure in such intimate crosstalk between clock and DDR factors may cause genomic instability and tumorigenesis.

Another link between DNA damage response and circadian clock relies on the regulatory feedback loop involving the tumor suppressor and checkpoint component p53 and PER2. In particular, wild-type p53 has been shown to negatively regulate PER2 expression. Mechanistically, Miki et al. demonstrated that wild-type p53 controls the circadian behavior of mice by competing with BMAL1:CLOCK for binding to the PER2 promoter, thereby repressing the expression of this latter^35^. In unstressed cells, temporal oscillations of p53 levels have been reported to be inversely correlated with PER2 levels, whereas, under stress conditions, accumulation of p53 levels to block PER2 transcription. Moreover, PER2 has been reported to form a trimeric complex with p53 and the oncogenic mouse double minute-2 homolog protein (MDM2), the p53’s negative regulator, thereby increasing p53 stability by blocking Mdm2-dependent ubiquitination and transcription of p53 target genes^36^. Based on the relevance of p53 in check-point signaling, such findings indicate that PER2 association with p53 might be a regulatory module that influences p53’s downstream response to genotoxic stress. Moreover, also the core clock component BMAL1 has been reported to bind to p53 promoter, thereby transcriptionally triggering the downstream tumor suppressor pathway^37^.

Therefore, such evidence reporting the molecular interactions between the core clock proteins and key cell-cycle regulators supports the notion of a link between circadian clock and cell cycle and that a circadian oscillator may establish temporal windows, thereby enabling or suppressing cell cycle transitions. Future investigations on how the biological clock governs cell proliferation and growth and on the potential implications for the onset of human diseases are warranted. Indeed, the temporal gating of cell division by the molecular clocks may be of relevance in the pathogenesis of cancer, since dysregulation of cell cycle represents a crucial driver of enhanced cell proliferative capacity. As a proof of concept, disruption of the regulatory loop linking p53, the cell cycle, and clock genes has been correlated to cancer in mouse models^38^. As an example, it has been demonstrated that a mutation (S662G) in PER2, which is known to be responsible for the familial advanced sleep phase syndrome, increased resistance to apoptosis and E1A- and RAS-driven oncogenic transformation, as well as affected tumorigenesis in p53^R172H/+^ mice, with a cancer-sensitized genetic background^38^. Noteworthy, the relative phases between p21 and Cyclin D expression profiles were significantly altered in PER2 allele mutant mouse embryonic fibroblasts, thus indicating that alteration of p21 phase, due to mutation, might impact on cell phase locking, thereby triggering cell cycle progression.

### Circadian clock, angiogenesis, and hypoxia

Although the role of circadian clock in controlling vascular development is largely unknown, evidence from the literature indicates that the core clock components participate in the regulation of angiogenesis, an essential process required for all tissue growth. Consistently, in developing zebrafish embryos, circadian regulatory genes, such as BMAL1 and PER2, have been reported to critically regulate vascular development, by modulating the expression of the vascular endothelial growth factor (VEGF)^28^. Disruption of the circadian clock by exposure to constant light and genetic manipulation of the core clock genes impaired developmental angiogenesis. Notably, the circadian regulator BMAL1 has been found to directly target and activate the promoter region of the *VEGF* gene *via* E-boxes, thereby promoting VEGF expression^28^. In addition, genetic deletion of BMAL1 impaired Notch-inhibition-induced vascular sprouting. Based on these data, it can be speculated that these findings unraveling mechanistic insights on the role of the circadian clock in controlling developmental angiogenesis may be also extended to other types of physiological angiogenesis (i.e. mammalian developmental angiogenesis), as well as to pathological angiogenesis in humans. In line with such hypothesis, the molecular clockwork has been found to regulate the expression of VEGF in hypoxic tumor cells^39^. In particular, the negative limbs of the molecular clock, i.e. PER2 and CRY1, have been reported to suppress the activity of VEGF promoter induced by hypoxia in the luciferase reporter gene analysis, thereby contributing to the circadian fluctuations of VEGF mRNA expression^39^.

Another master regulator of angiogenesis that has been shown to crosstalk with the molecular clock is the central regulator of oxygen homeostasis, i.e. the hypoxia-inducible factor 1α (HIF1α). Accordingly, HIF1α has been reported to bind to the E-Box and BMAL1, thereby promoting the expression of circadian genes^40–42^. Moreover, in U20S cells, E-box sequence in the promoter region of *HIF1α* gene as well as a direct binding of BMAL1:CLOCK complex to *HIF1α* have been observed^41^. As recently reviewed by O’Connell et al, 2020^43^, cell line- and tissue-specific bidirectional interactions between HIF and the core clock components have been found to regulate important biological functions. In this regard, Peek et al. demonstrated that the molecular clock is involved in the regulation of anerobic glycolysis *via* HIF1α signaling and that circadian control of HIF1α affects glucose metabolism in a context-dependent manner^44^. Indeed, while BMAL1^−/−^ liver displayed an enhanced anaerobic glycolytic gene expression^45^, BMAL1^−/−^ myotubes showed a decrease in anaerobic glycolysis, mitochondrial respiration, as well as transcription of HIF1α targets, including *VEGFa*^44^, thus suggesting a tissue-specific role of clock/ HIF1α interplay. Interestingly, the authors first demonstrated a time-of-day gating of HIF1α activity, with a greater activation during the active period (i.e. daytime in humans), suggesting that circadian control of HIF1α activity allows us to respond more robustly to hypoxia when demand for strenuous activity is likely to be higher.

Interestingly, in line with data showing a circadian/hypoxia pathway crosstalk, evidence from the literature indicates the acquisition of genetic signatures involving key players both in hypoxia and circadian pathways by long-term high-altitude populations living under hypobaric hypoxia conditions^46,47^. As an example, by using single-nucleotide polymorphism genotype data from Ethiopian populations, a strongest signal of selection was observed in the basic helix-loop-helix family member E41 (*BHLHE41*) gene that serves a major role both in the regulation of hypoxia-sensing pathway and circadian clock^47^. Of note, mutation in this gene has been associated with short sleep phenotype^48^.

Furthermore, hypoxia serves a key role in several diseases, including cancer, cardiovascular, lung, and metabolic diseases^49^. Therefore, the molecular mechanisms underpinning HIF and clock crosstalk both in physiological oxygen fluctuations and hypoxic conditions within different cells and tissues require further investigations. Moreover, studies are needed to understand the regulatory effects of the clock on the transcriptional hypoxic stress response, which is known to promote vessel growth by upregulating multiple pro-angiogenic factors. Such information may be of key relevance for understanding angiogenesis in pathological contexts such as vascular diseases and tumorigenesis.

### Circadian clock and immunity

Almost all the components of the immune system, involved in adaptive and innate immunity, have been observed to exhibit circadian variations (for a comprehensive review on the topic see^50,51^ For example, rhythmic oscillations occur in the trafficking of hematopoietic stem cells and leukocytes, in the expression of recognition receptors and their downstream signaling pathways, and finally in the production of cytokines and chemokines. As an example, Méndez et al demonstrated rhythmic Cxcl12 oscillations regulated by clock genes through the circadian regulation of noradrenaline secretion. In particular, the activation of β3-adrenergic receptors resulted in a reduction in Cxcl12 mRNA levels in bone marrow stromal cells, thus stimulating the mobilization of hematopoietic stem cells/progenitor^52^. Moreover, evidence from the literature indicates that the main components of immunity, including macrophages and natural killer (NK) cells, display a cell-autonomous circadian clock^53^. These endogenous clocks impose temporal gating across a range of functions, such as phagocytosis, cytokine/chemokine production, and antibacterial and antiviral activity^53^.

#### The interplay between circadian clock and inflammatory pathways

**Fig. 2:**
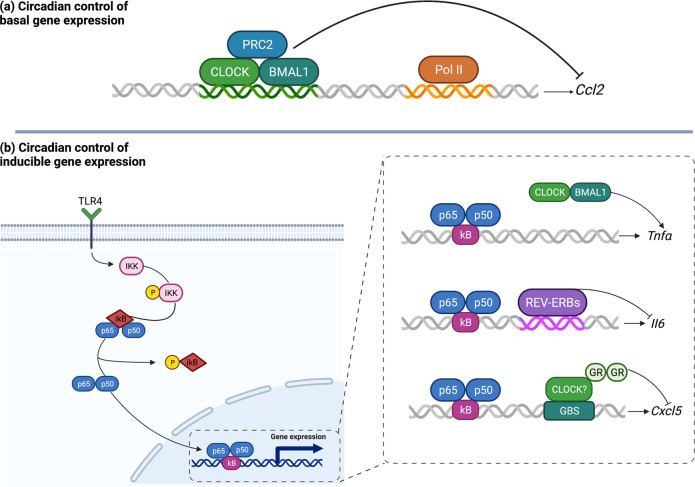
Circadian control of basal and inducible expression of inflammatory mediators in immune cells. **a** Circadian control of basal gene expression. Interaction between PRC2 and the CLOCK:BMAL1 complex rhythmically represses the expression of chemokine genes, such as *Ccl2*. **b** Circadian control of inducible gene expression. CLOCK acetylates the p65 subunit of NF-κB, thereby inducing the expression of TNFα. Moreover, recruitment of REV-ERB repressor complexes to inflammatory genes, such as *Il6*, rhythmically suppresses their expression. Finally, the cellular clock is fundamental for recruitment of GR complexes to the glucocorticoid binding site (GBS) on *Cxcl5* to repress its transcription. (BioRender.com has been used to create the figure)

The circadian clock has been suggested to serve a key role in the rhythmic regulation of basal inflammatory responses by temporally gating them. Accordingly, the circadian oscillators have been reported to prevent the synchronous activation of the different components of the immune system, by temporally limiting various aspects of the innate immunity, as well as the expression of inflammatory genes and the trafficking of innate immune cells to sites of inflammation, to specific phases of the circadian cycle (as comprehensively reviewed by Man et al^54^. As far as the clock-controlled expression of inflammatory mediators, BMAL1 has been demonstrated to generate basal oscillations in the expression of chemokines, such as CCL2, *via* interaction with the Polycomb repressor complex 2 (PRC2) in myeloid cells^55^. In particular, BMAL1 has been found to recruit PRC2 complex to repress chemokine gene expression (Fig. 2). Accordingly, its deletion resulted in an enhanced expression of *CCL2*, *CCL8*, and *S100a8* in monocytes and peritoneal macrophages^55^. Moreover, by using pharmacological and genetic approaches in human macrophages, REV-ERBα has been shown to act as a pivotal intermediary between the molecular clock and inflammatory pathways, by regulating the expression of genes involved in human innate immunity, such as *IL-6*^56^, and repressing *CCL2* gene expression directly through a RORE in the *CCL2* promoter region^57^ (Fig. 2). Furthermore, recruitment of REV-ERB repressor complexes to inflammatory genes has been reported to temporally repress their expression by inhibiting enhancer-specific transcription^58^.

Besides a direct regulation of the expression of inflammatory mediators by clock genes, another mechanism by which the molecular clock establishes a temporal gating of inflammation involves the glucocorticoid receptor (GR). Endogenous glucocorticoids regulate the circadian expression of a number of inflammatory cytokines through binding to the intracellular GR, which suppresses the expression of inflammatory mediators by interacting with GREs and by directly regulating transcription factors such as NF-κB, Activator protein-1 (AP-1) as transrepression^59^ (Fig. 2). It has been demonstrated that the molecular clock establishes a temporal gating of inflammation *via* GR. In this regard, Gibbs et al. reported a regulatory mechanism coupling the local molecular clock within the pulmonary epithelial cells with the GR–driven repressive effects on the expression of inflammatory chemokines^60^. Specifically, they found that, in bronchiolar cells, *CXCL5* expression was synergistically regulated by the local bronchiolar clock and the systemic repressive glucocorticoid signals of adrenal origin and that, in mice, genetic deletion of BMAL1 in bronchiolar cells impaired GR occupancy at the *CXCL5* locus and increased *CXCL5* expression despite normal corticosteroid secretion. These findings indicate that the recruitment of GR by the core clock gene BMAL1 may represent a mechanism to temporally repress the expression of inflammatory genes^60^, thus suggesting a key role for the adrenal axis in circadian control of inflammatory responses.

Furthermore, glucocorticoids have been shown to directly promote clock gene expression in both animal^61^ and human studies^62^. GREs have been described in the promoter regions of *PER* and dexamethasone has been observed to selectively induce *PER1* expression at concentrations consistent with the nighttime nadir of human cortisol^63^. Moreover, GR has been found to directly suppress REV-ERBα expression by interacting with CLOCK:BMAL1 complex in mouse liver^64^. In turn, CLOCK has been shown to physically interact with GR and to suppress its binding to DNA recognition sequences, by acetylating multiple lysine residues located in its hinge region in HCT116 and HeLa cells^65^. Such a result suggests that CLOCK:BMAL1 may antagonize biological actions of diurnally fluctuating circulating glucocorticoids. Lamia et al. also demonstrated that CRY mediate rhythmic expression of GR^66^. In particular, CRY 1 and 2 associate with a GRE in the phosphoenolpyruvate carboxykinase 1 (Pck1) promoter in a hormone-dependent manner, and dexamethasone-induced transcription of the *Pck1* gene has been found increased in CRY-deficient livers^66^.

Taken together, such evidence converges to indicate that the molecular clock directly exerts a regulatory control over inflammatory processes by finely tuning different intracellular mechanisms. However, this coupling between the circadian clock and inflammation has been suggested to be reciprocal, with also the circadian clock subjected to inflammation-driven reprogramming. In line with this hypothesis, Poolman et al. reported a significant circadian reprogramming due to chronic inflammation in patients with rheumatoid arthritis, with an increased circadian rhythmicity and expansion of the repertoire of rhythmic processes due to chronic joint inflammation^67^. These results reveal that, in chronic inflammatory disease, circadian mechanisms might be co-opted in order to set up a novel homeostatic oscillatory state, with an extensive reorganization of the circadian processes and *de novo* genesis of alternative cycling pathways. As an example, Wang et al. demonstrated that, in mice with dextran sodium sulfate (DSS)-induced colitis, colonic inflammation was associated with the dysregulation of clock genes (i.e. *NR1D1/REV-ERBα, CLOCK, BMALl1, PER2, CRY1, NPAS2, NR1D2/REV-ERBβ, RORα*, and *DBP*) expression^68^. However, whether this circadian reprogramming due to chronic inflammation may represent a disease-sustaining step is still unclear.

Notably, since circadian disruption has been correlated to the onset of several pathological conditions, understanding how the molecular clock intersects with inflammatory pathways, may provide novel insights into the etiopathogenesis of inflammatory conditions and reveal new therapeutic targets. Therefore, in the following sections, we will critically discuss the crosstalk between the circadian molecular clock, which introduces a temporal variable into cellular functions, and NF-κB transcription factor that have been reported to act as key molecular nodes integrating inflammation and circadian rhythmicity.

#### The crosstalk between NF-κB and the core clock components

Data from the literature provided evidence for an existing molecular crosstalk between the transcription factor NF-κB and the molecular components of the circadian clock. In this regard, a mechanistic framework for understanding the link between circadian rhythm, inflammatory processes, and NF-κB comes from the work by Narasimamurthy et al^69^. Indeed, the authors demonstrated that the absence of CRY protein(s), which act as transcriptional repressors, induce a constitutive increase in proinflammatory cytokines in a cell-autonomous manner. In particular, an increased phosphorylation of p65 at S276 residue due to increased protein kinase A (PKA) signaling activity has been found in Cry1^−/−^ and Cry2^−/−^ cells^69^. The absence of CRY has been hypothesized to release its inhibition on cAMP production, thereby increasing cAMP levels and PKA activation and, subsequently, PKA-mediated phosphorylation of the p65 subunit of NF-κB, leading to its constitutive activation. Furthermore, CRY mutation has been reported to promote the tumor necrosis factor α (TNFα)-initiated extrinsic apoptosis by interfering with NF-κB signaling in p53 mutant and oncogenically transformed cells^70^. Noteworthy, the core circadian clock activity has been shown to regulate the NF-κΒ pathway and apoptosis upon cytokine stimulation in p53-impaired cells, with the negative arm of the clock, i.e. CRY, inhibiting and the positive arm, i.e. BMAL1, stimulating apoptosis in this genetic background^70^. Accordingly, it was observed that, while stimulation with TNFα and IL-1β induced the transcription of NF-κB target genes, as well as its binding to their promoters, in *p53*^*KO*^ cells, the cytokine-driven transcriptional activation of NF-κB was abolished in *p53*^*KO*^ *Cry*^*DKO*^ cells. Interestingly, the effect of CRY mutation on the expression of NF-κB targets genes and NF-κB biding to their promoters was reversed by *BMAL1* down-regulation.

Besides a basal modulation of inflammatory processes, a rhythmic regulation of inducible gene expression has been suggested to rely on its crosstalk with NF-κB. In this regard, Spengler et al. demonstrated a direct molecular link between the transcription factors CLOCK and NF-κB. In particular, they found that, in the absence of BMAL1, NF-κB-driven transcription was upregulated by the core circadian protein CLOCK, whereas BMAL1 hindered the CLOCK-induced increase in the activation of NF-κB-regulated genes^71^. Furthermore, CLOCK was found in protein complexes with the p65 subunit of NF-κB, and CLOCK overexpression was associated with enhanced phosphorylated and acetylated transcriptionally active forms of p65^71^. In addition, NF-κB response was significantly reduced in *CLOCK*-deficient cells and in the tissues of *CLOCK*-knockout mice^71^, thus supporting the notion that CLOCK might act as a positive regulator of NF-κB transcriptional activity.

Another clock gene acting as a gatekeeper of inflammation is the nuclear hormone receptor REV-ERBα, a known repressor of NF-κB activity^57^. In murine macrophages, REV-ERBα has been shown to repress IL-6 expression indirectly through an NF-κB binding motif^72^. Consistently, it has been demonstrated to exert an anti-inflammatory activity in a murine model of colitis, by specifically repressing NF-κB and Nlrp3 expression, thereby downregulating Nlrp3 inflammasome activity^68^.

Furthermore, besides such evidence demonstrating a regulatory role of the molecular clock on NF-κB signaling pathway, it has been also observed that, vice versa, the activation of NF-κB directly impacts on the transcriptional activity of the core clock components. Accordingly, Maury et al. recently demonstrated that overactivation of NF-κB prevents BMAL1 binding to the promoter region of *PER2* and, consequently, its transcription in human omental fat^73^. In particular, BMAL1 has been found to bind in close proximity to NF-κB consensus motifs in human omental adipocyte precursors, revealing that IKKβ/NF-κB pathway may play a role in this repositioning. These findings indicate that inflammatory conditions associated with the overactivation of NF-κB may misalign the molecular clock, thereby altering the transcription of several target genes and contributing to metabolic inflammation through direct transcriptional reprogramming. As a proof of concept, obese mice with adipocyte-specific deletion of IKKβ displayed an improvement of adipose clock properties together with an improvement of metabolic inflammation^73^. In line with these findings, a previous study by Hong et al. reported that activation of NF-κB upon inflammatory stimuli markedly inhibited the clock repressors (i.e. *PER*, *CRY*, and *REV-ERB*) and relocated the clock components CLOCK/BMAL1 genome-wide to sites convergent with those bound by NF-κB^74^. These data indicate that NF-κB-driven transcriptional repression of the clock feedback limb might represent the triggering cause of circadian misalignment in response to inflammation and highlight a molecular mechanism through which immune activation may affect circadian rhythmicity. Moreover, conditional inactivation of IKKβ in mouse CNS and peripheral tissues impaired rhythmic activity behavior, thus revealing that IKKβ/NF-κB pathway controls circadian behavioral and molecular rhythms in vivo to preserve circadian homeostasis^74^. Therefore, it is tempting to speculate that basal level of NF-κB activity is fundamental for the maintenance of circadian homeostasis also in its latent state of immune signaling^74^.

### Circadian clock, metabolism, and redox homeostasis

In recent years, the existence of an extensive network of interactions between the circadian clock and the cellular redox state has emerged. As an example, reduced forms of the cofactors NAD(H) and NADP(H) have been shown to promote DNA-binding activity of BMAL1 and CLOCK, whereas their oxidized forms to hinder it, with minute changes in redox state strongly affecting the binding activity of circadian transcriptional^75^. Furthermore, another molecular redox-clockwork interaction involves Heme, a protein cofactor acting as a sensor of cellular redox state that has been reported to affect circadian control by binding to clock proteins. Consistently, it has been found to bind to REV-ERBs in a redox-state dependent manner^76^ to NPAS2, a CLOCK paralog, thereby modulating its DNA binding activity, as well as to inhibit binding of CLOCK to its E-box DNA target^77^. However, the mechanisms of regulation are largely unknown. Furthermore, the expression of nicotinamide phosphoribosyltransferase (NAMPT), a rate-limiting enzyme in the NAD^+^ salvage pathway, has been reported to be regulated by the BMAL1:CLOCK complex via the E-boxes present in its promoter in a time-dependent manner, thereby driving a rhythmic production of NAD^+ ^^78,79^. This latter activates NAD^+^-dependent histone deacetylases, such as sirtuin 1 (SIRT1), which has been shown to be fundamental for circadian transcription of several clock genes (e.g. BMAL1, PER2, and CRY1), as well as to bind BMAL1:CLOCK complex, and to promote the deacetylation and degradation of PER2^80^. Thus, since SIRT1 deacetylase activity relies on NAMPT-mediated NAD^+^ biosynthesis, SIRT1 has been hypothesized to link cellular metabolism to the circadian clockwork. Hence, NAD^+^ rhythmic production has been proposed to finely tune the daily cycles of energy storage and utilization, as well as to align such processes with the rest-activity cycle. In addition, a further crucial cellular energy sensor activated by a high AMP/ATP ratio, the AMP-activated protein kinase (AMPK), has been reported to mediate CRY and Casein kinases I phosphorylation, thereby modulating the negative molecular arm of the circadian clock by proteolytic degradation^81^. Then, the core clock gene BMAL1 has been shown to act as a negative regulator of the target of rapamycin complex 1 (mTORC1), which acts as a master switch between cell anabolic and catabolic programs, thereby suppressing anabolism^82^. Taken together, the above-mentioned examples demonstrate that redox state and metabolic pathways work in concert with molecular circadian timekeeping by exhibiting both input and output roles.

Besides these molecular interactions, circadian oscillation of redox state has been also linked to SCN neuronal excitability. In this regard, Wang et al. first demonstrated that redox state displays a daily rhythm in the brain^83^. By performing ratiometric redox fluorometry by two-photon microscopy of SCN organotypic slices, they observed a near-24-hour oscillation of redox state in SCN rat and mouse tissues^83^. Notably, circadian oscillation of global redox state in rat SCN was detected, with a marked oxidized state in the early night, when neuronal activity is at its lowest, compared to a reduced state during the daytime. The circadian redox rhythm was not observed in arrhythmic BMAL1^−/−^ mice, indicating that redox oscillations rely on a functional circadian clock^83^. Thus, such results suggest a potential mechanism by which the molecular clock influences SCN electrical activity. Moreover, since redox state has been described as a contributor to tissue-specific rhythmicity, it is tempting to speculate that circadian redox oscillations might extend beyond the SCN to other brain regions, as well as to all the excitable cells in the body, thereby allowing a nuanced fluctuation of cell excitability.

#### The role of Nrf2 as a central player integrating redox, immune and circadian signals

The nuclear factor (erythroid-derived 2)-like 2 (Nrf2) is a transcription factor regulating the expression of about 250 genes encoding a network of cooperating enzymes involved in endobiotic and xenobiotic biotransformation reactions, antioxidant metabolism, protein degradation, and regulation of inflammation^84^. By governing such complex transcriptional networks, Nrf2 coordinates a multifaceted response to various forms of stress, maintaining a homeostatic intracellular environment. Under unstressed conditions, Nrf2 is retained in the cytoplasm by its negative repressor Keap1 and rapidly subjected to ubiquitination and proteasomal degradation, mediated by the binding of Keap1 to the Cul3/Rbx1 E1 ubiquitin ligase complex^85^. After exposure to oxidative and/or electrophilic stimuli, Nrf2 is released from structurally modified Keap1 and translocates into the nucleus, forms a heterodimer with one of the small musculoaponeurotic fibrosarcoma (Maf) proteins, and activates the ARE-mediated expression of cytoprotective genes. Nrf2 is known to exert a pivotal role within the innate immune system, by specifically limiting inflammatory response via reactive oxygen species (ROS) suppression and preventing proinflammatory cytokine (i.e. interleukin-1β (IL-1β) and interleukin-6 (IL-6)) production through direct binding of Nrf2 to promoter regulatory regions^86,87^. Notably, a number of pharmacological and genetic studies demonstrates the existence of a functional crosstalk between NF-κB and Nrf2, with a range of cell-type- and tissue-dependent molecular interactions, fundamental for well-coordinated responses in inflammatory processes^88^.

Interestingly, several lines of evidence couple Nrf2 with the main core clock components. Rey et al. demonstrated that genetic or pharmacological inhibition of the pentose phosphate pathway (PPP) influenced circadian period length in a Nrf2-dependent manner^89^. In particular, they found that perturbation of PPP significantly increased Nrf2 DNA binding to *NR1D1* in U2OS cells, thus indicating that the Nrf2 activation may relay redox signals to circadian clock through *NR1D1*. Such results corroborate the hypothesis that Nrf2 represents a key nodal player between redox and circadian oscillations and provide a molecular mechanism explaining how redox imbalance, which is a key feature in many pathology (e.g. cancer, neurodegenerative and cardiovascular diseases), may disrupt circadian rhythmicity. A further study by Wible et al. substantiated these findings by showing that electrophilic or oxidative activation of Nrf2 signaling pathway influenced clock gene expression and circadian rhythmicity^90^. In particular, Nrf2 has been observed to repress its own transcription through the regulation of CRY2 and subsequent repression of CLOCK/BMAL1, thus forming an interlocking loop that integrates cellular redox signals with the core molecular circadian mechanism^90^.

Moreover, Nrf2 expression and activity have been reported to be under the circadian control in lung tissue. Accordingly, the circadian transcriptions factors CLOCK and BMAL1 exert a transcriptional control of *Nrf2* expression via E-box elements in the *Nrf2* gene promoter, thereby regulating Nrf2 protein accumulation in circadian manner and triggering a rhythmic expression of its antioxidant target genes^91^. Interestingly, the molecular clock has been revealed to regulate Nrf2 levels and activity also in innate immune cells. Indeed, Early et al. demonstrated that, in macrophages, BMAL1 modulated the mRNA expression of Nrf2 via direct E-box binding to its promoter and that its deletion disrupted Nrf2 activity and, consequently, stimulated the production of ROS and proinflammatory cytokines, such as IL-1β. Such evidence provides a mechanism by which the molecular clock controls aspects of innate immunity and highlight the crucial role of the core clock component BMAL1 in regulating the Nrf2-mediated antioxidant response in myeloid cells^92^.

Taken together, these results demonstrate that the *Nrf2* gene is directly regulated by the core clock via conserved E-box element in its promoter and also that Nrf2 transcriptionally regulates *BMAL1* and *CRY2* expression, thereby establishing a feedback loop and providing a possible molecular mechanism by which oxidative signals might input into the circadian clock.

## Circadian disruption: implication for human health and diseases

Circadian rhythms, when misaligned, become pivotal biological imperatives that might be challenged by environmental factors, social behaviors (e.g. exposure to artificial light, shift work, jet travel, irregular sleep, and eating schedules), as well as pre-existing pathological conditions. The subsequent circadian misalignment has been related to several pathologies, such as circadian rhythm sleep-wake disorders (e.g. insomnia, jet lag), cancer, obesity, arthritis, atherosclerosis, and mood disorders^93–96^. Moreover, although it will be not discussed herein, it cannot be discounted that also neuropsychiatric conditions, including, but not limited, depression, sundown syndrome in demented patients, and some very peculiar conditions (e.g. fatal familial insomnia) have been reported to be sensitive to external synchronization and correlated with alteration of circadian rhythm. In the following sections, we will specifically discuss circadian disruption in cancer and inflammatory diseases.

### Cancer

As discussed above, the molecular clockwork tightly regulates crucial cancer-related pathways, ranging from cell cycle progression to p53-mediated apoptosis. However, clock dysfunction has been either associated with pro- or anti-tumorigenic effects depending on model in a context-dependent manner, as recently reviewed by Stephenson et al, 2021^97^. Although the underpinning molecular mechanisms are not fully elucidated, epidemiological and clinical data provide evidence of a link between circadian misalignment/disruption and increased incidence of specific cancers. Notably, an increasing number of studies of carcinogenic risk factors suggested that disruptions in circadian rhythms play a more central role in tumor progression. Recent studies have revealed alteration in the status of post-translational modifications, such as phosphorylation and methylation, in the promoters of the core clock genes in cancerous tissues, leading to the deregulation of clock gene expression^98,99^. Based on such evidence, in 2007, shift work leading to a disruption in circadian rhythm has been listed by the International Agency for Research on Cancer (IARC) as a probable human carcinogen (Group 2A7).

Accordingly, preclinical data correlate the environmental disruption of the biological clock, due to shift work and light exposure at night, with hormone-dependent cancers, such as breast and prostate cancer^100–102^. As an example, the Nurses’ Health Study and case-controlled studies revealed that Norwegian nurses working night shifts for less than 30 years displayed a moderate increased risk for breast cancer, which was further increased upon working 30 or more years of rotating shift work^100^. Furthermore, evidence from the literature suggests an increased risk of prostate cancer in night-shift workers, further enhanced with a longer duration of shift work^101,103^.

Circadian rhythm alteration is also known to have a significant impact on the development of endocrine tumors^104,105^. Of note, compared to normal tissues, the expression of clock genes is altered in many solid human tumors and it has been identified an altered pattern in cyclic genes in human breast cancer cell line compared to normal breast epithelial cell line^106,107^. These observations point to a global reprogramming of circadian gene expression, rather than a disruption that may confer a physiological advantage to tumor cells and prompt to suggest that clock dysfunction could be considered a pro-tumoral event, thus indicating clock dysfunction as a potential hallmark of cancer. Indeed, in recent years, the hallmarks of cancer have been well established such as proliferative signaling, resisting cell death, evading growth suppressors, angiogenesis, activating invasion and metastasis, reprogramming of energy metabolism and evading immune destruction. Therefore, given the importance of clock-regulation, as described in the previous paragraphs in terms of cellular proliferation, in the regulation of the immune system and metabolic reprogramming, an impaired molecular clockwork may represent an emerging hallmark of cancer. Moreover, not only the circadian deregulation could be considered a hallmark of cancer, but also the circadian machinery has the capability itself to control and alter other cancer hallmarks. For example, as previously described, evidence from different eukaryotic model systems reveals a tight connection between circadian clock and cell cycle progression, DDR and DNA repair. Many genes crucial for cell cycle and division are under the control of clock genes, aberrantly expressed in several tumor tissues. Moreover, in order to maintain genome stability, the circadian clock system influences G1 to S phase transition (e.g. by regulating p21), preserves DDR, NER, and PARP1 activities, all of them dysregulated in cancer^30^. Indeed, the deregulation in any of these pathways has been found to trigger replication stress which, in turn, alters the DNA repair capacity, leading to genome instability and, finally, to cancer development. However, the signaling pathways and molecules inter-linking DDR, DNA repair and circadian clock genes are unclear.

Altogether, one of the main challenges in cancer field consists in translating the preclinical findings to clinics, therefore trying to unravel the relationship between clock genes expression, DNA repair capacity and treatment outcome. The comprehension of the expression levels of clock-controlled genes, proliferation markers, DNA repair and DDR genes derived from different cancer tissues represents an essential issue, since the common chemotherapy used to treat several cancers is mainly based on drugs that target cancer cell proliferation by inducing DNA break.

#### The circadian clock machinery regulates tumor immune microenvironment

It is now well accepted that the tumor microenvironment and, in particular, immune environment plays a crucial role in controlling tumor progression and, to date, several therapeutics (e.g. pembrolizumab; ipilimumab) are known to act on it. In this context, circadian machinery alteration not only affects tumoral cells but also the interaction between cancer cells and with other stromal components, thus affecting tumor development and metastatic colonization^108^. In support to this, it is now well accepted that most immune cell types, such as monocytes/macrophages^55,109^, dendritic cells^110^, neutrophils/MDSCs^111^, NK^112^, T and B cells^110,113^, have intrinsic circadian clock, and several of their characteristics/functions (e.g. plasticity, migratory capacity) are under circadian regulation.

The circadian aspect of many immune features is well documented in the contexts of infection and inflammation. However, the effect of circadian rhythms on tumor immune microenvironment is still nascent. Notably, tumor associated macrophages (TAMs)—the most abundant immune cells in solid cancers regulating immunosuppression, tumor growth, and metastasis formation—have a robust circadian rhythm and at least 10% of their transcriptional activity has been reported to be under circadian regulation^114^. Therefore, it is tempting to speculate that dysregulation of the circadian clocks in TAMs may have consequences on cancer progression and metastatization. For example, the deletion of BMAL1 in myeloid cells induced myeloid infiltration in the tumor in parallel with an increased inflammatory response (e.g. increase in CCL2–8, IL-1β, IL-6 levels)^92,115^. In addition, the silence of BMAL1 (e.g. BMAL1^−/−^ macrophages) favors the production of matrix metallopeptidase 9 (MMP-9)^116^ and the recruitment of CXCR2^+^ myeloid cells to the lungs^60^, events that are associated with the development of a pre-metastatic niche and metastatic dissemination/colonization. Moreover, long exposure to chronic jetlag of mice induced the development of spontaneous mammary tumors by an enrichment of TAMs and a reduction of anti-tumor cytotoxic CD8^+^ T cells in the tumor, therefore contributing to the development of immune-suppressive microenvironment a mechanism based on BMAL1-driven metabolic changes^117^. Thus, these studies suggest that the intimate interconnection between intrinsic circadian clock and cellular metabolism in the macrophages may play a critical role in shaping the tumor immune microenvironment.

Other key players of tumor immune environment are tumor associated neutrophils (TENs) that affect tumor progression by promoting angiogenesis, metastasis and by attenuating anti-tumor immunity^118^. The specific deletion of BMAL1 in TENs has been described to result in an impairment of homeostatic neutrophil clearance and enhanced migration to inflamed tissues, therefore suggesting a critical role of BMAL1/circadian machinery for the control of neutrophil immune responses^119,120^. In the context of tumor immune microenvironment, these findings suggest that circadian disruption potentially increases NETs formation, which has been shown to interfere with anti-tumor T and NK cell cytotoxicity^121^.

Among the other myeloid lineage, also dendritic cells (DCs), which are heterogenous antigen-presenting cells with a critical role in the activation of NL and T cell cytotoxicity, are affected by the clock machinery, even if the real mechanism at the basis is less understood. For example, BMAL1^−/−^ DCs had impaired capacity to induce Th1 immune responses due to the downregulation of several cytokines (e.g. IL-12, IL-23, or IL-27)^122^. Moreover, BMAL1^−/−^ DCs display also a reduced migration into the spleen, resulting in significantly reduced CD8 T cell expansion, a mechanism that is essential for the antitumor immunity response against cancer^123^. These data suggest that circadian disruption may alter DC-induced T cell responses either through the alteration of their cytokine production or through their migratory capacities.

Finally, also T and B lymphocyte seem to follow the circadian rhythm. For example, it has been demonstrated that their migration from the lymph nodes is regulated by the expression of CCR7 and S1PR1, a mechanism that is fine dependent on BMAL1. Furthermore, the secretion of cytokines (e.g. IL-2, IFNγ) from CD4^+^ T cells follows diurnal rhythms^113^. However, Treg cells do not seem to have a functional circadian machinery^124^. The general idea is that the circadian machinery may significantly impair the adaptive anti-tumor immune responses through the attenuation of T cell homing to the lymph nodes and the reduction of APC-T cell interaction with consequent reduction in T cell activation, an event essential for killing cancer cells.

Noteworthy, mutations in several components of the molecular clock have been linked to chemoresistance^125^. In this context, immunotherapy (e.g. anti-PD-L1, anti-CTLA4) has been used in recent years to overcome therapy resistance. However, many endocrine cancers, in which the clock genes are deregulated, are immunologically ‘cold’. Of note, data on the role of circadian machinery in anti-tumor immunity suggest a possible role of circadian machinery in the development of *cold tumors* through the accumulation of immune-suppressive cells in the microenvironment and through the regulation of the expression of PDL1 and CTLA4, two major targets of the current immunotherapy^126^. All these data suggest that circadian clock components also affect tumor escape mechanisms and, therefore, could be responsible for the induction of *cold tumors* and the failure for current immunotherapies.

Altogether the existing data suggest that clock genes are essential regulator of the anti-tumor immunity and the alteration of their expression leads to an immunosuppressive environment, leading to cancer progression, metastatization and the development of immune checkpoint escape mechanisms.

### Inflammatory diseases

Strong diurnal variations in the symptomatic expression and the severity of several chronic inflammatory diseases have been observed^127^. Given the role of the molecular clock in regulating immunity and inflammatory pathways under both homeostatic conditions and upon inflammatory challenge, deregulation of such rhythmic control has been suggested to promote an altered immune and inflammatory response. As an example, in mouse models of inflammatory arthritis, disruption of the molecular clock both in vivo, by constant light and genetic approaches, and in vitro, by pharmacological manipulation, altered clock gene expression in the joints, exacerbated joint inflammation, and enhanced arthritis score, thus indicating a direct role of the clock in disease progression^127^. However, while a robust link between circadian disruption and a number of inflammatory diseases is well documented, understanding of the underpinning molecular mechanisms is nascent. Indeed, although evidence from the literature discussed above reveals connections among the molecular clock, the immune system, and inflammation, as yet, little is known concerning the influence of the circadian clock on immune mechanisms underlying inflammatory disorders and several questions remain unanswered. First, circadian disruption and inflammation have been linked to these diseases independently; how an improper inflammatory response contributes to the onset of pathologies by dysregulating circadian rhythmicity and, vice versa, how circadian disruption triggers pathological inflammatory conditions remains largely unclear. Moreover, it is not known how a pathological condition in a given tissue may affect systemic circadian homeostasis in different tissues. Interestingly, Masri et al. demonstrated that lung adenocarcinoma contributed to the distal remodeling of circadian gene expression and metabolism in the liver *via* pro-inflammatory mediators, without affecting the hepatic core clock components^128^. Since the clock components of the liver were not altered, the authors speculated that the tumor may not function as a classical zeitgeber, but rather that it acts on the liver by rewiring circadian metabolic control, thereby dictating the pathophysiological dimension of a distal tissue^128^.

Therefore, further investigations are needed to decode the molecular mechanisms by which the circadian clock intersects with inflammatory signaling pathways in order to open new avenue for the treatment of inflammatory disorders.

## Time as a crucial dimension to medicine

Mounting evidence from the literature indicates that time represents a crucial dimension to medicine for establishing effective treatments in several pathological contexts. In particular, it is widely accepted that a breakthrough in the field of chronotherapy relies on establishing a circadian precision medicine based on treatments delivered in harmony with target physiology. Consistently, rhythmic oscillations ranging from drug absorption, distribution, metabolism, and excretion to the expression of drug targets have been shown to strongly impact on drug pharmacokinetics and pharmacodynamics. A proof of concept comes from clinical trials on patients affected by advanced stage ovarian cancer where the combination therapy, in which doxorubicin was administered at 6 a.m. and cisplatin given 12 h later, led to a fourfold increase in survival of patients, in comparison with patients treated with the same dose of both drugs at the same time (6 p.m)^129,130^. Moreover, the incidence of some pathological events has been reported to be time of the day dependent. Among the best-known examples, the incidence of myocardial infarction and stroke related to a rapid rise of blood pressure has been observed in the early morning^131,132^. Moreover, while rheumatoid arthritis patients experience joint stiffness and pain in the early morning, osteoarthritic patients experience pain towards the afternoon. In addition, asthmatic subjects are more prone to nocturnal worsening mainly due to the overnight recruitment of inflammatory cells into the airways^133^. In this regard, dosing of prednisone in the afternoon (3 p.m.) showed an increased efficacy in counteracting the inflammatory milieu and spirometric decline associated with nocturnal worsening of asthma, compared to its administration in the morning or later in the evening^133^.

Furthermore, Zhang et al. found that 56 of the top 100 best-selling drugs in the United States target products of a circadian gene^24^. Moreover, a meta-analysis of clinical trials comparing at least two different times of drug administration schedules revealed that, in the 75%, drug efficacy and/or toxicity relied on the time of the administration, across a number of conditions, such as hypertension, cancer, asthma, and arthritis^134^.

### Targeting circadian rhythm in pathology: pharmacological modulation of circadian machinery

Various therapeutic strategies targeting circadian rhythm are currently under investigation. In particular, besides ongoing clinical trials, not reviewed herein, testing the effects of behavioral and environmental modifications (e.g. light therapy, sleep interventions, and time-restricted feeding) on human health (Table 1), several attempts have been directed toward developing candidate drug molecules that specifically target the core clock components (e.g. REV-ERBs, RORs, PERs, CRYs), or other key regulators of the molecular oscillator (e.g. CK1), as detailed in Table 2.

**Table 1:** Environmental and lifestyle approaches in clinical trials

Environmental and lifestyle modifications	Disease indication	Purpose	Intervention/treatment	Clinical trial	Sponsor
Alteration in light exposure	Stroke; Sleep; Apnea Syndromes; Depression; Anxiety	Investigate the impact of exposure to ergonomic circadian light on physiological and mental parameters in stroke patients admitted for rehabilitation.	Device: Circadian Light luminaries	NCT02186392	Glostrup University Hospital, Copenhagen
	Alzheimer’s Disease	Determine effect and duration of timed therapeutic light, compared to control light on parameters of circadian rhythmicity, physiologic plasticity, sleep, and global function in women with Alzheimer’s Disease.	Device: Morning Simulated Sunlight Device: Non-therapeutic Red Light	NCT02502045	Yale University
	Depression-Postpartum	Establish the feasibility of light therapy for postpartum depression delivered via Re-Timer, demonstrate its preliminary efficacy, and clarify relationships between circadian shifts and mood changes using a novel, home-based circadian biomarker assessment paradigm (salivary dim light melatonin onset; DLMO).	Device: Light therapy	NCT02769858	University of Michigan
	Sleep Disturbances	Develop and evaluate a low-cost, minimally obtrusive device that delivers individualized light therapy to adults with early-awakening insomnia.	Other: Blue light Other: Red light	NCT01855126	Rensselaer Polytechnic Institute
	Seasonal Affective Disorder	Develop a new light therapy device with more blue light (blue enriched polychromatic light) to treat seasonal affective disorder.	Device: Original Energy Light Device: Original Energy Light prototype	NCT01048294	University Medical Center Groningen
	Concussion; Mild Post-Concussion Symptoms; Sleep Problems	Verify whether bright light therapy may be helpful in improving the sleep of patients with a recent history of mild traumatic brain injuries and if may also have other mood elevating effects.	Device: wavelength-1 bright light Device: wavelength-2 bright light	NCT01747811	University of Arizona
Time-restricted feeding	Overweight Obesity; Weight Loss	Examine the influence of timing of eating on sleep patterns, physical activity, and self-reported feelings of appetite control.	Behavioral: Eat majority of calories in the morning Behavioral: Eat the majority of calories in the evening	NCT02204735	The University of Tennessee, Knoxville
	Obesity; Abdominal Dyslipidemias; Insulin Resistance; Blood Pressure; Weight Loss	Develop and hone dietary counseling approaches for time restricted eating for RD’s in a clinical practice paradigm and collect data on testing this intervention compared to standard dietary counseling approaches for cardiometabolic health.	Behavioral: Time Restricted Eating Behavioral: Standard Cardiometabolic Health Diet	NCT03527290	University of California, Irvine
Change in sleeping time	Traumatic Brain Injury	Monitor sleep efficiency, post traumatic amnesia, agitation and cognitive function and examine relationships among them.	Behavioral: Sleep Hygiene Protocol Behavioral: Standard of Care	NCT02838082	Craig Hospital
	Breast Cancer	Verify whether improvement in sleep in women night shift workers will have a positive impact on biological and behavioral risk factors associated with breast cancer and quality of life.	Behavioral: Sleep intervention	NCT02609373	University of British Columbia
Scheduled activity	Type 2 Diabetes; Insulin Independent	Compare the efficacy of morning and afternoon HIIT in lowering blood glucose values in participants with type 2 diabetes.	Other: morning HIIT -->afternoon HIIT Other: afternoon HIIT -->morning HIIT	NCT03553524	Karolinska Institute
	Alzheimer’s Disease	Characterize objective sleep parameters and behavioral symptoms of sleep-wake disturbance, and biological indicators of diurnal HPA axis activity in a sample of community residing older adults with AD; Examine the effects of timed and planned activities on subjective and objective characteristics of sleep, behavioral symptoms, and HPA status; Evaluate measurement approaches in home-dwelling AD patients.	Behavioral: Timed Planned Activity Behavioral: Home Safety and Education Program	NCT01920672	Johns Hopkins University
Combination of: changing in light exposure; feed and physical activity	Critical Illness; Sleep Deprivation; Respiratory Failure; Sleep Disorders; Circadian Rhythm	Determine whether the sleep and circadian rhythms of critically ill patients undergoing mechanical ventilation can be improved through practical strategies that can be employed at the bedside.	Behavioral: Sleep and circadian rhythm promotion Behavioral: Usual care	NCT01284140	Brian Gehlbach
	Healthy Night Shift Workers	Investigate the effects of 12 weeks of randomized timed light therapy or timed physical exercise as a chronotherapeutic lifestyle intervention on markers of central and peripheral circadian rhythms and cardiometabolic function in healthy night shift workers.	Other: Intensive light therapy Other: Exercise	NCT01767181	Universitätsklinikum Hamburg-Eppendorf
	Fatigue in HIV	Determine the overall feasibility of a behavioral intervention for managing fatigue among older adults with HIV infection. Estimate effect sizes for group differences at 1, 2, and 3 months on five dimensions of fatigue.	Behavioral: Sleep and Rhythm Intervention Behavioral: Dietary Modifications	NCT02126007	University of California, San Francisco
	Premenstrual Dysphoric Disorder	Examine the effects of co-administered wake therapy followed by light treatment on mood, and secondarily on circadian rhythms, to test the hypothesis that critically timed chronotherapy improves mood by correcting phase disturbances in melatonin and sleep in women with Premenstrual Dysphoric Disorder.	Other: LWT + AM BWL Other: EWT + PM BWL	NCT01799733	University of California, San Diego
	Depression; Depressive Disorder, Major; Depression, Unipolar; Depression, Moderate	Determine whether altering the pattern of one’s sleep and having light therapy can speed up the treatment of depression.	Behavioral: Wake and Light Therapy Behavioral: Sleep and Light Therapy	NCT03405493	King’s College London
	Major Depressive Disorder; Bipolar Disorder	Replicate previous findings that sleep deprivation results in marked improvement in depression symptoms, as well as to test whether concurrent treatment with Light Therapy and Lithium are successful in locking in and maintaining therapeutic effects in both bipolar and unipolar depressed subjects.	Behavioral: Wake Therapy Device: light box Drug: Lithium	NCT01431573	New York State Psychiatric Institute

Date of consultation: 12/12/2021 (Clinical trials: https://clinicaltrials.gov/)

*RD* registered dietitian, *HIIT* high intensity interval training, *HPA* hypothalamic/pituitary/adrenal, *AD* Alzheimer’s disease, *LWT+Am BWL* Late Wake Therapy plus morning bright light, *EWT* *+* *PM BWL* Early Wake Therapy plus evening bright light

**Table 2:** Development of small molecules targeting the core clock machinery

Target	Drugs	Mechanism of actions	Effects	Experimental model	Pathological context	Refs.
REV-ERBα/β	GSK4112	REV-ERBα/β agonist	–Inhibition of cell-proliferation, by preventing transition from G1 to S phase. –Increased expression of the proliferation inhibitor (p27) and suppression of the expression of the proliferation-promoting factor Cyclin D and β-catenin. –Induction of apoptosis, with an increase in Bax mRNA levels and Caspase-3 and a decrease in Bcl-2 levels.	Mouse 3T3-L1 adipocytes	Obesity	^148^
			–Reduction of ALT and AST plasmatic levels. –Improvement of liver condition and the survival rate in Jo2-insulted mice. –Inhibition of hepatocyte apoptosis, with a reduction of caspase-3 and caspase-8 activities.	Wild-type and Jo2 treated C57BL/6 mice	Liver injury	^149^
			–Inhibition of LPS-induced phosphorylation of IκK, thereby blocking p65 nuclear translocation and suppressing the expression of proinflammatory cytokines, such as IL-6 and TNFα.	BV2 cells and Male C57BL/6 mice	Neuro-inflammation; neurodegenerative diseases; psychiatric disorders	^150^
			–Protection of ventral midbrain neurons from LPS-induced microglial activation-induced damage.			
	SR9001 SR9009	REV-ERBα/β agonists	–Reduction of the expression of GSC markers (e.g. *OLIG2* and *SOX2*), supporting negative transcriptional regulation of key GSC targets. –Decreased GSC cell proliferation and the expression of genes involved in glycolysis, TCA cycle, and lipid metabolism.	Derived Human Glioblastoma Stem Cells	Glioblastoma	^145^
			–Reduction of GBM growth. –Induction of apoptosis and downregulation of autophagic genes (Ulk3- Ulk1, Beclin1, and Atg7).	C57BL/6	Glioblastoma	^137^
			–Increase in apoptosis in NRAS-induced naevi and repression of autophagic gene expression (Ulk3, Ulk1, Beclin1, and Atg7).	C57BL/6 and Tyr-NrasQ61K mice	Melanoma, NRAS-induced naevi	^137^
	SR8278	REV-ERBα/β antagonist	–Improvement of microglial uptake of fibrillar amyloid-β (fAβ_1‐42_) –Microglia polarization toward a phagocytic M2‐like phenotype with increased P2Y12 receptor expression, thereby enabling the phagocytosis of Aβ aggregates.	Murine‐immortalized microglial BV‐2 cells 5XFAD and REV‐ERBα knockout mice	Alzheimer’s disease	^151^
			–Improvement of renal condition, by decreasing renal damage and cell death. –Diminution in renal malondialdehyde, iron levels and mitochondrial damage. –Increase in renal GSH and Gpx4 levels.	Wild-type C57BL/6 mice treated with folic acid	Acute kidney injury	^152^
	ARN5187	Dual inhibitory activity toward both REV-ERBs and autophagy	–Disruption of lysosomal function, blockade of autophagy at the late stage, and reduction of cancer cell viability. –Inhibition of REV-ERB mediated transcriptional regulation.	Breast cancer BT-474 cells	Breast cancer	^153^
ROR	Nobiletin	RORs agonist	–Improvement of strengthening circadian amplitude, causing an enhanced efficiency in physiological performance, greater stimuli range and sensitized response. –Reduction of body weight in WT mice fed with high-fat diet thanks to a reduction in fat mass and white adipose cell size. –Improvement of oxygen consumption with a switch from lipid-biased metabolism to a more balanced contribution from all major macronutrients. –Improvement in lipid and glucose metabolism, with a reduction in plasmatic level of insulin, total triglyceride and cholesterol.	Diet-induced obesity (DIO) mouse model using both WT and clock-disrupted *Clock*^*Δ19/Δ19*^ mice	Metabolic syndrome (obesity)	^154^
			–Promotion of healthy aging in metabolically stressed mammals. –Increase in the activation of MRCs genes, leading to an improvement of mitochondrial function (i.e. increase of ATP production and reduction of ROS levels).	C57BL/6 mice and C2C12 myoblast cells	Aging and metabolic syndrome	^155^
	SR1001	RORγt inverse agonist	–Reduction of retinal inflammation in diabetes. –Reduction of IL-17, TNF-α and VEGF serum levels. –Reduction of leukostasis. –Decrease in diabetic degenerative capillaries.	C57BL/6 mice WT and treated with STZ	Diabetes	^156^
			–Slowdown the onset and clinical severity of EAE. –Reduction of IL-17, IL-21 and IL-22 mRNA levels. –Reduction in T CD4^+^ cells population.	Wild-type and EAE C57BL/6 J; Hep-G2 cells	Autoimmune diseases (multiple sclerosis)	^139^
			–Reduction of IL-17A and IL-17F mRNA levels in mouse blood cells and prostate tissues. –Reduction of proliferation, angiogenesis and inflammatory cell infiltration, as well an increase in apoptosis in mouse model of prostate cancer.	*Pten*-null mice	Prostate cancer	^157^
	SR3335	RORα selective partial inverse agonist	–Inhibition of RORα target genes expression in HepG2 involved in hepatic gluconeogenesis. –Reduction of glucose plasmatic levels in mouse model.	Diet induced obese (DIO) C57BL/6 mice and HepG2 cells	Diabetes	^158^
			–Reduction of ILC2 cell population proliferation. –Inhibition of rynovirus-induced mucus metaplasia in immature mice. –Reduction of IL-13 and mucus-related mRNA levels.	BALB/c mice affected by rynovirus	Respiratory disease (asthma)	^159^
	SR1078	RORs agonist	–Reduction of repetitive behavior, associated with autism. –Increased expression of autism-associated RORα target genes in mouse brain.	BTBR T + Itpr3tf/J (BTBR)	Autism	^160^
			–Reduction of aerobic glycolysis and down-regulation of biosynthetic pathways in vitro. –Reduction of PDK2 mRNA levels and inhibition of the phosphorylation of pyruvate dehydrogenase. –Reduction of proliferation in hepatoma in vitro and in a xenograft model in vivo.	Hepatoma cell lines and n a xenograft model in vivo	Hepatoma	^161^
	Digoxine and derivates	RORγ inverse agonist	–Inhibition of IL-17 expression in human CD4^+^ T cells. –Reduction of IL-17a protein levels in naïve mouse CD4^+^ T cells.	Mouse and human CD4^+^ T cell culture	Inflammatory and autoimmune disease.	^141^
			–Reduction of the arthritic score and arthritis incidence. –Histological analyses showed a reduction in inflammatory cell infiltration and cartilage loss in mouse ankles. –Decrease in the expression of proinflammatory cytokines. –Reduction of the number of Th17 and increase of Treg population in mice with collagen-induced arthritis.	DBA/1J mice treated with bovine type II collagen and CD4^+^ T cells obtained from spleen of DBA/1J mice	Autoimmune arthritis	^162^
			–Reduction of Th17 cells in PBMCs. –Decrease in IL-1β, IL-6, IL-17, and IL-23 protein levels in supernatant of digoxin treated PBMCs.	PBMCs of RA patients	Rheumatoid arthritis	^163^
	SR2211	RORγ inverse agonist	–Inhibition in growth and proliferation of prostate cancer cells. –Promotion of apoptosis.	Doxorubicin-resistant prostate cancer C4-2B cells	Prostate cancer	^164^
			–Reduction in the expression and production of inflammatory cytokines in Th17 cells. –Reduction of LPS-driven IL-1β, IL-6, and TNFα expression in vitro. –Reduction of joint inflammation in mice with CIA.	DBA/1 J mice and Th17 cells and RAW 264.7 cells	Collagen-induced arthritis	^165^
CRY1/2	KL001	Stabilization of CRYs by inhibiting FBXL3-mediated ubiquitination and degradation of CRY proteins	–Significantly enhanced glucose clearance in a dose-dependent manner. –Reduction of fasting blood glucose.	db/db mouse model of diabetes and (DIO) C57BL/6J mouse model	Diabetes	^142,143^
			–Inhibition of glucagon-dependent expression of PCK1 and G6pc genes without influence their basal expression. –Specific inhibition of glucagon-mediated activation of glucose production.	Primary hepatocytes prepared at ZT9-11 from fed mice (C57BL/6*)*		^144^
			–Decrease in *OLIG2* and *SOX2* mRNA levels. –Inhibition GSC proliferation due to the increase in CRY1 overexpression, leading to a decrease in Myc expression.	Human GSCs and NSG immunocompromised mice	Glioblastoma	^145^
	SHP656	CRYs stabilizers	–Reduction of GSC cell number –Increase in survival of mice bearing two different patient-derived GSCs	Human GSCs and NSG immunocompromised mice	Glioblastoma	^145^
	KS15	CRYs inhibitor	–Increase in Per2 and REV-ERBα expression and reduction of BMAL1 mRNA levels in MCF-7 cells. –Reduction of Cyclin D1, c-Myc e Bcl-2 mRNA and protein levels and increase in p53, Bax, and Wee1 levels, thus suggesting the ability of compound to inhibit cell cycle. –Reduction of proliferation in MCF-7 cells. –Improvement of chemosensitivity.	MCF-7 cells	Breast cancer	^166^
CK1	IC261	CK1δ/ε inhibitor	–Inhibition of HCC with an accumulation of cells in G2/M phase. –Increase in the number of apoptotic cells and increase in cleaved-PARP protein levels. –Inhibition of tumor growth in mice bearing HCC tumor xenografts –Reduction in both tumor volume and weight.	HCC cell lines and BALB/c nude mice bearing HCC tumor xenografts	Hepatocellular carcinoma	^167^
	PF-670462	CK1ε and CK1δ Inhibitor	–Reduction in chemotaxis, invasion and communication with stromal cells in primary CLL cells and in all major subtypes of CLL. –Decrease in the leukemic cell’s accumulation in the peripheral blood and spleen. –Improvement of life expectancy of mice affected by CLL.	Primary cells isolated from peripheral blood of CLL patients and Eµ-TCL1 mouse model	Chronic lymphocytic leukemia	^168^
			–Normalization of hippocampal proteomic alterations (e.g. proteins involved in synaptic plasticity, cytoskeletal organization, mitochondrial function and elaboration of amyloid precursor protein) associated with AD-like pathology. –Improvement of cognitive disturbances (increase of memory and reduction of anxiety) associated with AD. –Restoration of circadian rhythms.	3xTg-AD Mice	Alzheimer’s Disease	^169^

*BAX* Bcl-2 associated X, *Bcl-2* B-cell lymphoma 2, *ALT* alanine aminotransferase, *AST* aspartate transaminase, *Jo2* anti-fas antibody, *LPS* lipopolysaccharide, *NF-κB* nuclear factor kappa B, *IκK* inhibitor of NF-κB alpha kinase, *IL-6* interleukin 6, *TNFα* tumor necrosis factor α, *GSC* glioblastoma stem cells, *OLIG2* oligodendrocyte lineage transcription factor 2, *SOX2* SRY-box transcription factor 2, *TCA* tricarboxylic acid, *GBM* glioblastoma, *Ulk3-Ulk1* unc-51 like kinase 3-1, *Atg7* autophagy related 7, *NRAS* NRAS proto-oncogene, GTPase, *fAβ*_*1-42*_ fibrillar amyloid-beta, *GSH* glutathione, *Gpx4* glutathione peroxidase 4, *DIO* diet-induced obesity, *MRCs* mitochondrial respiratory chain complexes, *ATP* adenosine triphosphate, *ROS* reactive oxygen species, *IL-17* interleukin 17, *VEGF* vascular-endothelial growth factor, *STZ* streptozotocin, *IL-21* interleukin 21, *IL-22* interleukin 22, *EAE* experimental autoimmune encephalomyelitis, *ILC2* innate lymphoid type-2 cells, *RV* rinovirus, *IL-13* interleukin 13, *Muc5ac* mucin 5AC, oligomeric mucus/gel-forming, *BTBR* mouse model of autism spectrum disorders, *A2bp1* ataxin 2-binding protein 1, *Cyp19a* cytochrome P450 aromatase genes, *ITPR1* inositol 1,4,5-trisphosphate receptor type 1, *PDK2* pyruvate dehydrogenase kinase 2, *IL-1 β* interleukin 1-β, *IL-6* interleukin 6, *IL-23* interleukin-23, *CIA* mouse collagen induced arthritis, *PBMCs* peripheral blood mononuclear cells, *RA* rheumatoid arthritis, *CIA* collagen-induced arthritis, *PCK1* phosphoenolpyruvate carboxykinase 1, *G6pc* glucose-6-phosphatase catalytic subunit, *SCN* suprachiasmatic nucleus, *GSK3* glycogen synthase kinase-3, *HCC* hepatocellular carcinoma, *CLL* chronic lymphocytic leukemia, *AD* Alzheimer’s disease (AD)

Small molecules directly targeting the clock proteins offer the potential to directly manipulate endogenous circadian rhythm to improve clock-regulated output processes, as well as to treat diseases associated with clock misalignment (for a comprehensive review on the topic see^135^. As an example, in in vivo studies, synthetic REV-ERBα/β agonists, such as SR9009 and SR9011, showed remarkable effects in metabolic diseases, cancer, as well as mood disorders^136–138^. In this regard, Solt et al. demonstrated that, in diet-induced obese mice, the administration of a REV-ERBα/β agonists reduced obesity by decreasing fat mass and significantly ameliorating dyslipidaemia and hyperglycemia, thus indicating a potential applicability for the treatment of metabolic diseases^136^. In addition, Sulli et al. observed that the pharmacological activation of REV-ERBs by SR9009 and SR9011 selectively induced cytotoxicity in leukemia and several solid tumors with different tumorigenesis drivers^137^. In particular, SR9009 and SR9011 induced apoptosis in cancer cells and oncogene-induced senescent cells, including melanocytic naevi, without affecting the viability of normal cells or tissues, by inactivating two key cancer hallmarks, such as *de novo* lipogenesis and autophagy^137^.

Furthermore, several RORs synthetic ligands have been optimized and tested in in vivo studies in different pathological contexts, including autoimmune disorders^139,140^. Consistently, RORα/RORγ ligands (i.e. SR1001) have been reported to suppress the differentiation of T-helper cells that produce interleukin-17 (Th17 cells), crucial effector cells implicated in the pathology of numerous autoimmune diseases, and to reduce cytokines expression, thereby alleviating autoimmune disease symptoms in animal models of multiple sclerosis^139^. In line with such results, also non-toxic digoxin-like synthetic derivatives (20,22-dihydrodigoxin-21,23-diol and digoxin-21-salicylidene) have been shown to specifically block IL-17 expression in human CD4^+^ T cells by inhibiting RORγt activity^141^.

Another potential therapeutic route is based on the use of CRY-stabilizing compounds^142,143^. In this regard, the CRY1- and CRY2-stabilizing carbazole small molecule KL001 was first identified in a cell-based screen to lengthen period and reduce amplitude^144^. Structural studies further demonstrated that KL001 binds to the FAD-binding pocket of CRY and interferes with its recognition by FBXL3, thus hindering CRY ubiquitination and the consequent proteasomal degradation^144^. Interestingly, analysis of The Cancer Genome Atlas (TCGA) revealed that higher levels of mRNAs encoding the circadian clock repressor CRY2 were associated with improved survival in patients with glioma, thus supporting the hypothesis that the use of CRY-stabilizing compounds might be a promising strategy for glioblastoma therapy in humans^145^. Within this context, among the developed CRY stabilizers, the most promising compound SHP656, derived from KL001, has been found to specifically inhibit the growth of patient-derived glioblastoma stem cells in vitro, without impacting on differentiated glioblastoma cells or non-malignant epilepsy-derived neural cells^145^. Moreover, in the mouse model of glioblastoma, treatment with SHP656 reduced tumor growth and prolonged mouse survival^145^. Recently, isoform-selective compounds (i.e. KL101 and TH301, selectively stabilizing CRY1 and CRY2, respectively) have been developed, thereby offering valuable tools to test in order to assess the potential similarities and differences of CRY1 and CRY2 selective stabilization^146^.

Based on the growing number of clock modulators with promising pharmacokinetic properties and efficacies in different mouse models of diseases, extending their preclinical efficacy to human trials represent a major future challenge to assess the therapeutic potential of circadian manipulation. In particular, given the role of the core components in controlling key aspects of immune response (e.g. immune cell development, function, and trafficking), the pharmacological modulators of circadian proteins may represent a novel approach to reprogram the tumor microenvironment by promoting anti-tumor immunity and, consequently, impinging upon cancer immune surveillance.

## Conclusions

**Fig. 3:**
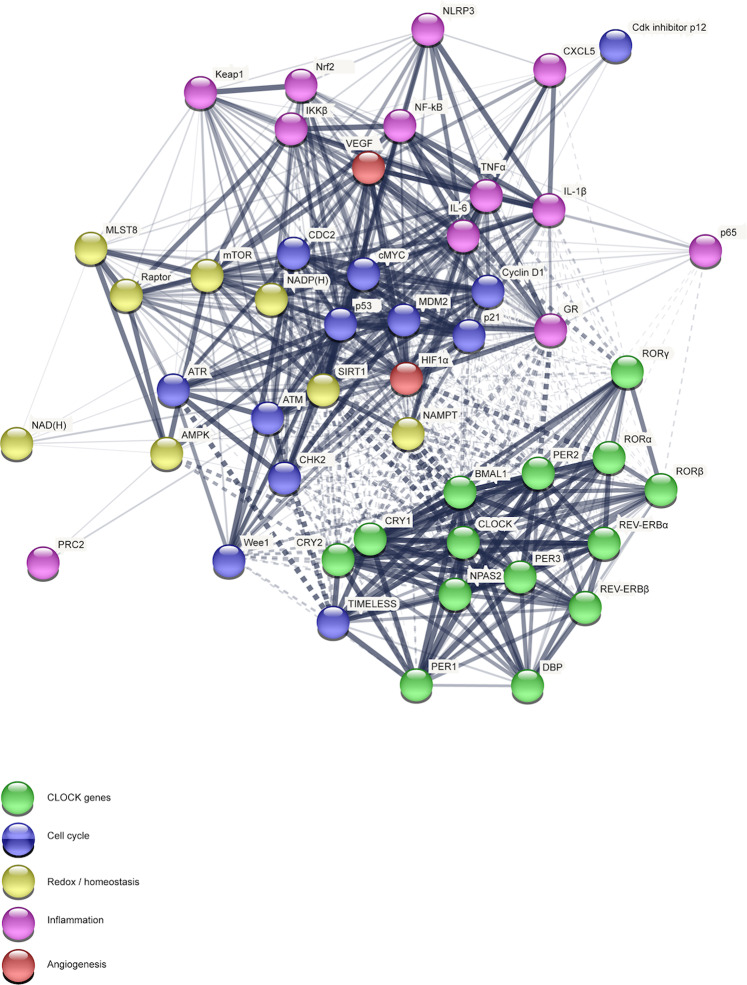
Protein–protein interaction (PPI) network between the core clock components and clock-related proteins. STRING Protein–Protein Interaction database (Ver 11.5)^147^ has been used to build the PPI network. The network contains 48 nodes and the edges represent the crosstalk between the core clock components and proteins belonging to other key intracellular pathways. Line thickness reflects the strength of data support for protein-protein interaction that derives from databases, experiments, or based on computational predictions. The network is clustered based on a specified “MCL inflation parameter” and on a customized clustering coefficient of 0.300. The different biological processes investigated are shown in the network according to the color legend

The core clock components represent key molecular players temporally gating many intracellular signaling pathways, ranging from cell growth, DNA repair and DDR, angiogenesis, apoptosis, metabolism, redox state, to immune and inflammatory processes. In this regard, based on the complex scenario depicted by evidence from the literature discussed above, we applied a model-based network approach for the *Homo Sapiens* proteome recapitulating the mechanistic connection between the molecular clock and the biological pathways herein investigated (Fig. 3). The protein-protein interaction (PPI) network was built starting from a small group of proteins selected from the literature showing a significative evidence to be functionally correlated with the circadian molecular clock. Subsequently, the output generated by STRING (Ver 11.5)^147^ was manually filtered in order to clearly summarize the interconnection between the core clock components and different cellular pathways.

A detailed analysis of the network reported in Fig. 3 shows an intricate and highly interconnected PPI network between the core clock components and several proteins belonging to other key intracellular pathways. In particular, there is peculiar distribution of the proteins into the network in which the clock genes are uniformly grouped forming a dense cluster. The clock-related proteins belonging to different pathways strongly interact each other and concomitantly with all the core clock components, increasing significantly the complexity of the PPI network. Taking into account the complexity of these multiple intra- and interconnections, the circadian clock emerges as a crucial regulator of key physiological processes and its misalignment as potential triggering factor and/or pathological outcome in several human diseases, such as cancer and inflammatory diseases. Although a detailed description of PPI is beyond the aim of our review, such network partially recapitulates the peculiar crosstalk and close association between the intrinsic biological clock and the intracellular signaling pathways described above, with relevant implications in the therapeutic management of diseases. Furthermore, the PPI network shown in Fig. 3 is a clear example of how different intracellular pathways, which appear to be clearly grouped and separated from each other, are functionally interconnected both between the different groups and also between the different members of each group.

Overall, to deep inside the molecular and mechanistic knowledge of circadian pathways, their relationship in pathophysiological processes, as well as the development of novel chronotherapeutics, represent an intriguing and transversal medicine challenge.
